# High performance, ion-exchange chromatography of amino-acids in biological fluids using Chromakon 500- performance of the apparatus

**DOI:** 10.1155/S146392468500044X

**Published:** 1985

**Authors:** Luc Cynober, Colette Coudray-Lucas, Frédéric Ziegler, Jacqueline Giboudeau

**Affiliations:** Laboratoire de Biochimie A Hôpital Saint Antoine 184, rue du Fg. St. Antoine Paris Cedex 12 75571 France


**Journal of Automatic Chemistry**/*Journal of Clinical Laboratory Automation*, **Vol. 7, No. 4 (October–December 1985), pp. 201–203**

### High performance ion-exchange chromatography of amino-acids in biological fluids using Chromakon 500 – performance of the apparatus

**Luc Cynober, Colette Coudray-Lucas, Frédéric Ziegler and Jacqueline Giboudeau**

*Laboratoire de Biochimie A, Hôpital Saint Antoine, 184, rue du Fg. St. Antoine, 75571 Paris Cedex 12, France*

### Introduction

The separation and quantitative measurement of amino-acids by ion-exchange chromatography has been in use for many years [1]. However, the value of this method is limited by the time required for the analysis: 6 to 23 h. Other methods, such as gas chromatography, give results more rapidly, but do not allow the separation of all amino-acids [2]. The development of cation-exchange resins, capable of resisting high pressure, considerably shortens the time required to perform an analysis. In this study the performance of the Chromakon 500 (Kontron, Switzerland) with a cation-exchange resin (Kontron AS-70 [3]) is evaluated; 39 amino-acids and derivatives can be separated in less than 130 min (170 min including the regeneration time).

### Material and methods

All the reagents necessary for the ion-exchange chromatography were supplied by Merck (Darmstadt, FR Germany). Stabilized lyophylized serum (set Hw) was provided by Bio Merieux (Charbonnières les Bains, France). The 40 amino-acid standard solution was supplied by Pierce and was supplemented with glutamine (final concentration 250 µmol/l) from Calbiochem (San Diego, California, USA). Amino-acids were separated on the Chromakon 500 equipped with an automatic injection loop, a 15 cm column packed with Kontron AS 70 resin (diameter 7 µ) and with five citrate buffers (see table 1). A simplified diagram of the apparatus is given in figure 1. The elution program was a slightly modified version of that given by Kontron (table 2) with modifications which improved the separation of the following amino-acids: cysteine and methionine, cystathionine and isoleucine, 3- methylhistidine and anserine. Detection was performed by colorimetry at 570 + 440 nm with the ninhydrin reaction.

The apparatus was coupled to a Shimadzu CR 1 B integrator and amino-acid concentrations were calculated by the method of pic areas. Prior to analysis, stabilized serum was deproteinized by sulphosalicylic acid (50 mg/ml serum) and half diluted in buffer 1.

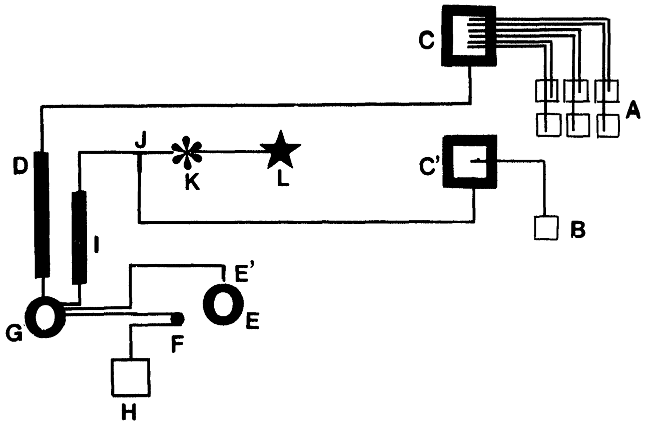

*Figure 1. Simplified diagram of the apparatus. Where A = buffer reserves; B = ninhydrin reserve; C = piston pumps (C = buffer, C' = reagent); D = buffer purification column; E = sample circlet; E' = sample needle; F = peristaltic pump; G = injection loop; H = rinsing solution reserves (methanol); I = cation-exchange column; J = eluate/ninhydrin T-junction; K = oven; L = photometer.*

### Results and discussion

The stability of retention times was studied by 10 consecutive injections of the calibration solution. Amino-acids were adequately separated (a typical chromatogram is shown in figure 2). Retention times are constant: CVs ranged from 0.1 to 1.5% (table 3) and are rather better than those provided by liquid HPLC [4]. When the apparatus is stopped, it is interesting to note that a 1 h equilibration time with buffer 1 is required before performing the first analysis in order to obtain constant retention times.

*Table 1. Composition of buffers.*

| | 1 | 2 | 3 | 4 | 5 |
|---|---|---|---|---|---|
| Lithium hydroxide monohydrate (g) | 5·05 | 5·45 | 8·4 | 8·4 | 8·4 |
| Citric acid monohydrate (g) | 17·4 | 17·4 | 17·4 | 17·4 | 14·7 |
| Lithium chloride (g) | 0 | 0 | 0 | 16 | 35 |
| Chlorhydric acid (ml) | 19 | 15 | 20 | 8 | — |
| Phenol (ml) | 2 | 2 | 2 | 2 | 2 |
| Methanol (ml) | 70 | 50 | — | — | — |
| Purified water, quantity for 1 l | | | | | |
| The buffers were filtered, degassed, and left for 12 h before adjusting the pH to: | 2·60 | 3·10 | 3·75 | 4·0 | 5·25 |

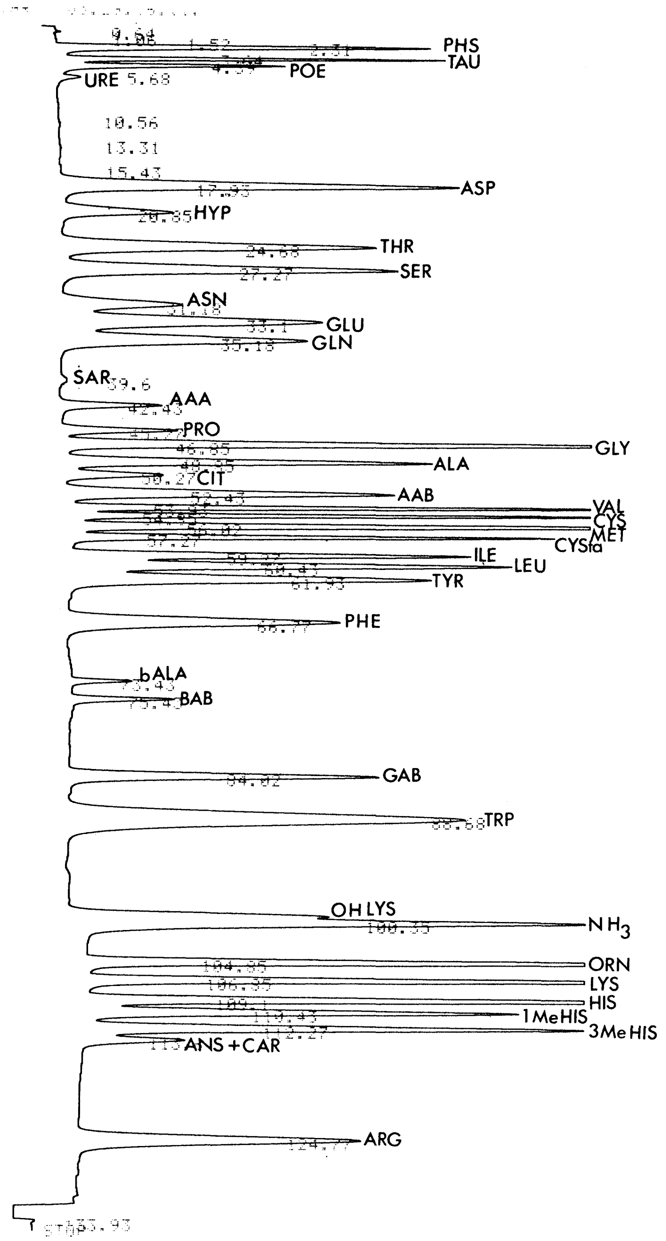

*Figure 2. A typical chromatogram obtained with the standard solution supplemented with glutamine. Where PHS = phosphoserine, TAU = taurine, POE: phosphoethanolamine, URE = urea, ASP = aspartic acid, HYP = hydroxyproline, THR = threonine, SER = sérine, ASN = asparagine, GLU = glutamic acid, GLN = glutamine, ALA = alanine, SAR = sarcosine, AAA = alpha aminoadipic acid, PRO = proline, GLY = glycine, VAL = valine, CYS = cysteine, MET = methionine, CYSTa = cyslathionine, ILE = isoleucine, LEU = leucine, TYR = tyrosine, PHE = phenylalanine, bALA = β alanine, BAB = β aminoisobutyric acid, GAB = γ aminobutyrique acid, TRP = tryptophane, OH LYS = hydroxylysine, $NH_3$ = ammoniac, ORN = ornithine, LYS = lysine, HIS = histidine, 1 MeHIS = 1-methylhistidine, 3 MeHis = 3 methylhistidine, ANS + CAR = anserine + carnosine, ARG = arginine.*

Linearity and detection limit assays were performed by dilutions of the standard (2500 or 1250 µmol/l according to the amino-acids, 500, 250, 125, 50, 25, 10 and 5

*Table 2. Elution of amino-acids.*

| | | |
|---|---|---|
| Passage time of buffer 1 | 0 | min |
| pH of buffer 1 | 2·60 | |
| 1st temperature | 35 °C | |
| Cooling stopped | 34 | min |
| Passage time of buffer 2 | 11 | min |
| pH of buffer 2 | 3·10 | |
| 2nd temperature | 62 °C | |
| 2nd temperature at | 37 | min |
| Passage time of buffer 3 | 43 | min |
| pH of buffer 3 | 3·75 | |
| Passage time of buffer 4 | 66 | min |
| pH of buffer 4 | 4·00 | |
| Passage time of buffer 5 | 97 | min |
| pH of buffer 5 | 5·25 | |
| 3rd temperature | 70 °C | |
| 3rd temperature at | 88 | min |
| Time buffer 6 (regeneration) | 127 | min |
| Column cooling time | 135 | min |
| Return-time buffer 1 | 138 | min |

N.B.: Times given are from the beginning of the analysis.

µmol/l). The reaction was linear to a concentration of at least 1250 µmol/l, which is higher than usual plasma and urine concentrations for all amino-acids given that samples are half-diluted before analysis. The detection limit was less than 5 µmol/l, except for glutamate and glutamine (10 µmol/l) and phenylalanine (25 µmol/l).

Repeatability assays performed by 10 consecutive measurements of amino-acid contents of a lyophylized serum (HW Unitrol) gave good results (table 3). The CV of all amino-acids was under 5%, except for phenylalanine (8·3%). Reproducibility assays performed with HW Unitrol serum measured in 10 different series, gave a mean coefficient of variation of 10.2%. The comparatively poor results concern proline (17·6%). These results are comparable to those obtained by liquid-liquid HPLC [4] and classic ion-exchange chromatography [5], but are not quite as good as those obtained by gas chromatography [6].

In conclusion, this apparatus gives precise results for physiological amino-acids in a reasonably short time, so the use of this high-performance ion exchange chromatography is very attractive.

A useful feature is that the analysis program can be modifed at all times. Similarly, the creation of new programs is very simple, for example the program can be shortened if one is particularly interested in the amino-acids. Breakdowns are infrequent, provided the apparatus is regularly maintained. Special attention should be paid to cleaning the air filter supplying cooling air to the electronic circuitry. The flow of ninhydrin must be stopped when passing the regeneration buffer because the liberation of certain very ninhydrin-sensitive substances leads to the formation of crystals which block the system.

The blockage of the separation and purification columns can be avoided by inverting them every two months. Column-life is very satisfactory: the same column has been in use for more than 18 months. Finally, it is worth noting that the equipment is more reliable if it is in continuous use.

*Table 3. Stability of retention time, repeatability and reproducibility assays.*

| Amino-acid | Retention time x̄ ± SD (minutes) | CV% | Repeatability ($N$ = 10) x̄ ± SD (μmol/l) | CV% | Reproducibility ($N$ = 10) x̄ ± SD (μmol/l) | CV% |
|---|---|---|---|---|---|---|
| PHS | 2·17 ± 0·02 | 0·9 | 25 ± 0·9 | 3·6 | 29 ± 2 | 8·4 |
| TAU | 3·72 ± 0·02 | 1·5 | 91 ± 2·3 | 2·5 | 72 ± 15 | 11·1 |
| ASP | 17·83 ± 0·21 | 1·2 | 58 ± 1·1 | 1·8 | 40 ± 5 | 12·5 |
| HYP | 20·63 ± 0·16 | 0·2 | <5 | | <5 | |
| THR | 24·55 ± 0·15 | 0·6 | 77 ± 2·8 | 3·6 | 78 ± 7 | 9·3 |
| SER | 27·27 ± 0·15 | 0·6 | 56 ± 2·3 | 4·1 | 70 ± 7 | 9·5 |
| ASN | 38·46 ± 0·27 | 0·7 | <5 | | <5 | |
| GLU | 39·53 ± 0·33 | 0·8 | 107 ± 2·8 | 2·6 | 137 ± 14 | 10·2 |
| GLN | 40·26 ± 0·24 | 0·6 | 29 ± 1·0 | 3·4 | 72 ± 8 | 11·1 |
| PRO | 49·85 ± 0·34 | 0·7 | 75 ± 1·8 | 2·4 | 90 ± 16 | 17·6 |
| GLY | 51·45 ± 0·36 | 0·7 | 304 ± 2·5 | 0·8 | 333 ± 31 | 9·3 |
| ALA | 53·13 ± 0·48 | 0·9 | 306 ± 5·2 | 1·7 | 271 ± 22 | 7·9 |
| CIT | 54·45 ± 0·38 | 0·7 | 53 ± 2·0 | 3·5 | 55 ± 5 | 10·0 |
| VAL | 59·83 ± 0·45 | 0·8 | 232 ± 4·5 | 1·9 | 213 ± 20 | 9·4 |
| CYS | 62·99 ± 0·36 | 0·6 | <5 | | <5 | |
| MET | 63·81 ± 0·35 | 0·5 | 31 ± 0·4 | 1·3 | 29 ± 3 | 10·3 |
| ILE | 65·51 ± 0·85 | 1·3 | 93 ± 2·0 | 2·2 | 78 ± 8 | 10·0 |
| LEU | 67·21 ± 0·36 | 0·5 | 199 ± 2·8 | 1·3 | 162 ± 13 | 8·2 |
| TYR | 68·25 ± 0·11 | 0·2 | 41 ± 1·8 | 4·4 | 43 ± 5 | 11·6 |
| PHE | 72·83 ± 0·45 | 0·6 | 63 ± 5·0 | 8·3 | 62 ± 5·5 | 8·9 |
| TRP | 91·80 ± 0·52 | 0·6 | 39 ± 0·7 | 1·8 | 36 ± 3 | 8·5 |
| ORN | 106·27 ± 0·50 | 0·5 | 114 ± 1·5 | 1·3 | 86 ± 10 | 11·6 |
| LYS | 108·51 ± 0·43 | 0·4 | 133 ± 2·3 | 1·7 | 118 ± 15 | 12·7 |
| HIS | 110·79 ± 0·21 | 0·2 | 86 ± 1·4 | 1·6 | 78 ± 7·5 | 9·6 |
| ARG | 126·73 ± 0·06 | 0·1 | 169 ± 4·6 | 2·7 | 140 ± 11 | 7·6 |

### References

1. MOORE, S. and STEIN, W. H., *Journal of Biological Chemistry*, **211** (1954), 893.

2. CYNOBER, L., BLONDE, F., NGUYEN DINH, F., GERBET, D. and GIBOUDEAU, J., *Annales de Biologie Clinique*, **41** (1983), 33.

3. HUGHES, G. J. and WILSON, K. J., *Journal of Chromatography*, **242** (1982), 337.

4. BIGGS, H. G. and GENTILCORE, L. J., *Clinical Chemistry*, **30** (1984), 851.

5. CASTETS, J. C., PARVY, P. and HUANG, Y., *Annales de Biologie Clinique*, **39** (1981), 45.

6. YAMAMOTO, S., KIYAMA, S., WATANABE, Y. and MAKITA, M., *Journal of Chromatography*, **233** (1982), 39.

**FOOD ADDITIVES & CONTAMINANTS**

Now in its third volume, this Taylor & Francis journal contains original research and review articles relating to the detection, determination, occurrence, persistence, safety evaluation and control of naturally occurring and man-made additives and contaminants in the food chain.

Contributions include aspects of the chemistry, biochemistry and bioavailability of these substances, their metabolites or reaction products; advances in analytical methodology; and factors affecting levels of potentially toxic compounds which may arise during processing, packaging and storage.

Recent articles include:

Sulphiting agents in foods: some risk/benefit considerations
*R. Walker, UK*

The absence of rhamnose in human urine following the ingestion of gum karaya *(Sterculia)*
*A. W. J. Anderson* et al.,

Chromium in foods and the diet
*G. A. Smart and J. C. Sherlock, UK*

Naturally occurring oestrogens in foods — a review
*K. R. Price and G. R. Fenwick, UK*

Interaction between plastics packaging materials and foodstuffs
*W.-D. Bieber, K. Figge and J. Koch, FR Germany*

*Sample copies from Taylor & Francis.*

